# Overcoming Challenges Resulting From COVID-19: New York State’s Creating Healthy Schools and Communities Initiative

**DOI:** 10.5888/pcd17.200232

**Published:** 2020-07-09

**Authors:** Tamara Vehige Calise, Amelia Fox, Amanda Ryder, Laura Rios Ruggiero

**Affiliations:** 1 JSI Research & Training Institute, Inc, Healthy Communities, Boston, Massachusetts

## Abstract

“Upstream” interventions that increase access or reduce barriers to healthy foods and opportunities for physical activity — referred to as policy, systems, or environmental strategies — are central to encouraging and supporting healthy behaviors that prevent chronic disease at a population level. However, they are complex and challenging to execute, especially during coronavirus disease 2019 (COVID-19), and efforts to build practitioner capacity are warranted. In this commentary, we describe a user or human-centered design (HCD) capacity-building approach to support practitioners in accomplishing the goals of the New York State Creating Healthy Schools and Communities (CHSC) initiative. This approach has been especially helpful during COVID-19, as it enables support to be responsive to practitioners’ constantly changing needs. Given that CHSC is a project specific to New York State and that the efforts of the Obesity Prevention Center for Excellence were tailored to obesity prevention, more research and evaluations should be conducted to better understand how the use of HCD could support practitioners addressing other complex public health issues in the United States.

**Summary**

**What is already known on this topic?**

Increasing access or reducing barriers to healthy foods and opportunities for physical activity are central to encouraging and supporting healthy behaviors that prevent chronic disease at a population level. While strategies to increase access or reduce barriers are generally difficult to implement, the COVID-19 pandemic has added challenges.

**What is added by this report?**

The New York State Creating Healthy Schools and Communities initiative used a human-centered design (HCD) that enabled support to be responsive to practitioners’ constantly changing needs during the pandemic.

**What are the implications for public health practice?**

More research and evaluations should be conducted to better understand how the use of HCD could support practitioners addressing other complex public health issues in the United States.

## Introduction

Factors beyond health care, including those that are often outside the scope of traditional public health activities (eg, health education), impact health (1). “Upstream” interventions that increase access or reduce barriers to healthy foods and opportunities for physical activity — referred to as policy, systems, or environmental strategies (PSEs) — are central to encouraging and supporting healthy behaviors that prevent chronic disease at a population level (2–4). However, they are complex and challenging to execute (5,6).

Practitioners positioned to implement PSEs include those working in local departments of public health, transportation, and planning, as well as education and community-based organizations (7). Often working together, they must address an array of factors at the levels of social systems, communities, and organizations (8). However, they may be unaware of the health problem and solutions (eg, educational organizations may not focus primarily on health); lack the capacity to act with a systems perspective (eg, work across sectors toward health goals); or struggle with politics, differing organizational protocols, vocabularies, and funding (9–12). To prioritize and successfully implement PSEs, practitioners and their respective organizations must have capacity, including resources and networks in and beyond the communities they represent (12–16).

Capacity building is necessary to support the implementation of PSEs (10,11,17). It includes activities to develop or improve the knowledge, skills, commitment, collaboration, structures, and systems at the individual, organizational, and community levels (18,19). Various models have been implemented to build this multilevel capacity (10,18,20) including funders or contracted agencies that provide one-on-one consultation and web-based learning options, develop materials and resources, and facilitate training opportunities (17). For example, the Centers for Disease Control and Prevention (CDC) and its partners provided support to state-level practitioners implementing obesity prevention initiatives (11); the National Association of Chronic Disease Directors and others were contracted to support communities funded by Action Communities for Health, Innovation, and Environmental Change (ACHIEVE) (18); and the Missouri Department of Health and Senior Services provided assistance to local public health agencies implementing Building Communities for Better Health (20).

## Creating Healthy Schools and Communities

Creating Healthy Schools and Communities (CHSC) is a 5-year (2015–2020), coordinated, multisector initiative of the New York State Department of Health (NYSDOH) with the goal of reducing major risk factors of obesity, diabetes, and other chronic diseases in 85 high-need school districts and associated communities (N = 266). CHSC practitioners (CPs) work with individuals, schools, government, businesses, and other groups to share ideas, plan, and act to improve access to healthy foods and opportunities for physical activity. Since its launch, NYSDOH has contracted with the Obesity Prevention Center for Excellence (OPCE), whose sole charge has been to build CHSC capacity, both in terms of the practitioners and the communities in which they work. The OPCE capacity-building model draws largely on the principles of user or human-centered design (HCD) (21,22), in that staff work jointly with NYSDOH, CPs, and other beneficiaries to co-create capacity-building activities to ensure successful PSE implementation. Most importantly, the 4 iterative phases of HCD (discovery, definition, design, and implementation) enable OPCE’s capacity building to be responsive to CPs’ constantly evolving needs.

In the discovery phase, OPCE interacts regularly with NYSDOH and CPs and conducts evaluation surveys and annual assessments to identify practitioner assets, needs, motivations, and concerns. OPCE accounts for diverse perspectives and provides an array of solutions for CPs. The information collected throughout the continuous discovery phase is used to determine where OPCE can support practitioners individually, as well as identify where it can support synergies, collaboration, and opportunities to leverage resources across the state. The input of NYSDOH and CPs ultimately drives the design and implementation processes.

The ultimate goal of CHSC is to strengthen food systems, increase opportunities for physical activity, and promote wellness policies and practices in worksites and schools. Unfortunately, New York State has been the epicenter of the coronavirus disease 2019 (COVID-19), which was first diagnosed in the United States in January 2020 (23). CPs and community partners continue to experience challenges such as school and worksite closures and, more broadly, issues of food insecurity in their communities, which have forced them to redirect their work while ensuring that the original goals of CHSC are achieved. 

The purpose of this commentary is to present how the use of an HCD approach has enabled OPCE to return repeatedly to the context, emotions, needs, and desires of its intended beneficiaries during these uncertain times to strengthen and sustain their capacity to implement PSEs in their communities.

## Pre-COVID-19 Capacity Building – Focus on CHSC Practitioners and Organizational Levels

In the first few years of CHSC, NYSDOH and CPs requested support that was specific to the 6 CHSC strategies (Figure). OPCE provided technical assistance in the form of resources, individual consultations, in-person meetings, collaborative brainstorming calls, collaborative learning communities, monthly newsletters, an online collaboration and resource library, and virtual trainings. For example, OPCE facilitated a brainstorming call where CPs talked about recruiting worksites to adopt food service guidelines and worked with NYSDOH to create a worksite wellness recognition program as a way for CPs to engage worksites in their efforts to increase access to healthy foods in the community. Over the past few years, the capacity-building support also included resources on engaging local policy makers, effective communication and messaging, and skills necessary for ensuring PSE success. OPCE also worked with national partners to tailor technical assistance to meet the needs of CPs and their local partners. One example includes the partnership between OPCE and America Walks (americawalks.org) to design and implement the New York State Walking College (https://americawalks.org/walkingcollege/). In this initiative, CPs and their planning and transportation partners received tailored assistance in expanding local leadership capacity and multi-stakeholder partnerships for walkable communities by learning about strengthening municipalities’ commitment to Complete Streets policies and implementation plans, traffic calming pop-up projects, Vision Zero (https://visionzeronetwork.org/) policies and goals, and leveraging additional funding for Complete Streets design and construction.

**Figure:**
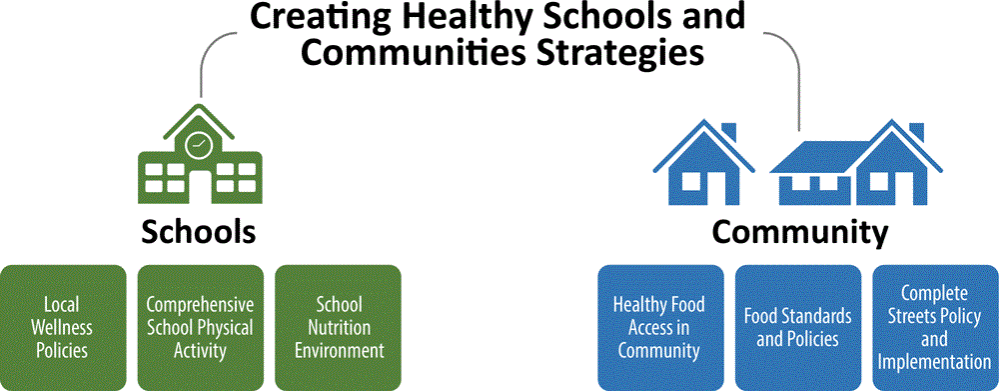
Schematic diagram of the Creating Healthy Schools and Communities initiative, New York State Department of Health, 2015–2020.

## And Then There Was COVID

There is no “business as usual” during COVID-19, which continues to bring with it a host of issues that affect the ability of CPs to conduct their work. Beginning in March 2020, OPCE has assessed the needs and challenges of CPs and their partners. In addition to needing support with implementing PSEs during school and community closures, many reported personal feelings arose, from frustration to empowerment, and there was recognition of the importance of self-care during the pandemic. In an article on self-care and parents, Coyne et al (24) describe several evidence-based practices that align with those taken by OPCE to support CPs, including delivery of self-care in small, manageable “doses,” and strength in numbers.

### Small acts of self-care

OPCE offered opportunities for CPs to engage in small acts of self-care by providing space for them to share stories and support to one another. Virtual trainings start with self-care questions such as “What is one thing you have done to support your own wellness in the last week?” or “What has made you laugh most recently?” CPs are asked to share aloud or via the call’s chat function, and this exchange is followed by conversation. To provide regular “brain breaks” for CPs, OPCE offers weekly 10-minute virtual Zumba Fitness sessions (Zumba Fitness, LLC) led by one of the technical assistance leads.

### Strength in numbers

As CHSC was entering its fifth year, CPs wanted to build their capacity and that of their school partners to ensure the sustainability of their efforts. OPCE worked collaboratively with leadership experts to design and implement a Leadership Academy, which was launched at the end of January 2020, just before COVID-19. A total of 54 participants were enrolled (17 CPs and 37 school staff of various roles [ie, physical education teacher, guidance counselor, principal, nurse, wellness coordinator, and assistant superintendent]).

The Leadership Academy focuses on adaptive leadership and includes individual coaching sessions and action learning groups. To date, 42 people (28 school staff and 14 CPs) participated in 1 or more coaching sessions (mean, 3 sessions). The remaining 12 Leadership Academy enrollees declined or did not participate. Action learning groups expand individual support through group interactions in which participants receive and provide feedback on PSE implementation. Originally, OPCE and leadership experts created teams of people in various roles from New York State. However, CPs and their school partners expressed concerns because of school closures and other challenges related to COVID-19. As a result, OPCE changed the structure from monthly scheduled calls to weekly open drop-ins. Nine drop-in action learning groups were held in April and May 2020, with 7 to 13 people participating per call (mean, 10 participants). In total, 43% of those who signed up for the Leadership Academy have participated (9 school staff and 14 CPs) in 1 or more action learning group sessions (mean, 4 sessions). Although a formal evaluation has yet to be conducted for the Leadership Academy, OPCE has received informal feedback from CPs and coaches that the opportunity has been invaluable during the pandemic. One CP reported, “I’m very appreciative [of the Leadership Academy opportunity] and love my coach. . . . We talk about our challenges, successes, and everything that comes up.” Another stated, “I have done the coaching calls and have been to almost every weekly ALG [Action Learning Group] call. . . . I find them to be so useful.” Another reported that she has “learned so much talking to other people around the state,” and that “it [the Leadership Academy] came at the right time [during COVID-19].”

Even without a crisis, practitioners like to hear from others with similar experiences (11), and peer-to-peer interaction is especially helpful during times of stress (24). OPCE hosted a virtual “solution room” where CPs presented challenges to their colleagues for feedback and suggestions. Topics included how to 1) support a virtual school wellness committee; 2) proceed with required grant assessments; 3) sustain engagement with populations in which religion or other barriers may prevent them from having or using technology; 4) encourage safe physical activity, including walking and biking; and 5) use platforms to sustain engagement with school and community partners. This format was well received by CPs, one of whom stated, “I feel some comfort in the fact that many of us are going through similar issues and challenges . . . and that we came together to provide some solutions to some challenges.” As issues of equity, diversity, and inclusion are being elevated nationally and throughout New York State, CPs are now requesting opportunities to discuss social injustice with their peers; OPCE is currently (June 2020) using the HCD approach in an attempt to meet this need.

## Sustaining Efforts and Achieving CHSC Goals Post COVID-19

Communication efforts can help garner support and change public opinion, raise awareness of solutions, and build capacity among diverse sectors and constituencies (15,16). CPs have worked for years to increase awareness of CHSC and establish relationships with school and community-based partners. They empathize with their partners and wanted to support them during this pandemic and the resulting uncertainty. Several content-specific needs identified during COVID-19 included encouraging students and employees to be physically active during the day, helping households to access food, and creating safe outdoor spaces that support social distancing.

Since the start of the pandemic, organizations throughout the United States have developed many resources. OPCE sorted through the information and created an online database most relevant to CHSC where CPs can also share resources. As of this writing, 60 resources are listed, including free guided physical activity for adults, live-streamed recesses and physical activity toolkits for parents and teachers, state department of education updates, and nutrition guidance through the Supplemental Nutrition Assistance Program.

CPs report sharing these resources with their respective school and community partners who also disseminate them among their constituents. One CP reported sharing the resources through her virtual community engagement teams to support administrators, teachers, and students. Another CP used the database and skills she learned from action learning groups to facilitate a virtual wellness workshop that brought partnering school districts together. She collaborated with an educational organization to offer continuing teacher and leader education credits for staff who attend the meetings, which has helped to meet a staff need. The first virtual wellness workshop was so successful that partners agreed to meet again to discuss updating the wellness policy and the completion of the triennial assessment.

In addition to content-specific needs, CPs identified several technical areas, such as virtual engagement, that they felt could help develop and maintain partnerships and related efforts during COVID-19. OPCE responded quickly to enhance capacity to virtually engage stakeholders by developing and disseminating a guide that included video conferencing and production, communications, team management, and external engagement platforms. The guide was followed by a brainstorming call to see how implementation of these resources was going and where additional support might be needed.

Given the attention to the impact of chronic disease and the risk it poses for severe illness, CPs want to increase awareness of maintaining a healthy lifestyle and the PSEs they have helped to implement across the state. OPCE developed a “toolbox” with a compilation of resources to support communication efforts and delivered a webinar entitled Elevate Your Design Skill Set: Tips, Tricks, and Tools. Both of these resources supported integrated and strategic communications efforts through the development of effective messaging and strengthened graphic design capabilities. In addition, OPCE created resources that could be tailored and used for easy dissemination, including a 1-page template for sharing CHSC-related resources. CPs share the template electronically to reinforce their willingness and availability to support partners during COVID-19.

OPCE also supported CPs to increase awareness of chronic disease prevention by creating “bite-size” messages that reinforced health and CHSC for CPs to share through social media. Messages included “People of all ages with chronic conditions such as diabetes and heart disease are at highest risk if they contract the coronavirus. Increasing access to healthy foods and opportunities for physical activity is a critical component of preventing chronic diseases. Since 2015, CHSC has worked to create environments that are supportive of overall health and wellness in 245 communities across New York” and “COVID-19 has more people relying on biking and active transportation for both physical activity and a means of transport. The need for widespread, sufficient bike infrastructure has never been more apparent. . . . CHSC has worked with communities across New York to expand bike infrastructure at over 205 sites.”

The support OPCE has provided to CPs and their partners has helped not only to successfully implement many PSEs but also to support their response to the emerging needs of their schools and communities.

### Building on partnerships to increase access to healthy foods during the pandemic

For the past 5 years (starting in 2015), CHSC-funded organizations have worked closely to help small retailers, bodegas, and food pantries stock and sell healthy, affordable foods. These efforts have expanded access to healthy foods by enhancing the food supply chain. Before COVID-19, the North Country Healthy Heart Network helped the Joint Council for Economic Opportunity secure funding to install 2 additional greenhouses equipped with hydroponic systems where fresh produce could be harvested for the mobile farmer’s market. The market has 21 stops in 10 communities and sells to a local distributor that serves local schools, hospitals, and jails. When stay-at-home orders went into effect and food pantry supplies were low, the Joint Council for Economic Opportunity redirected its products to stock pantries and emergency food packages for those in need, reaffirming the critical role of local food retailers and pantries in the larger food system, especially in lower-income communities that have limited access to full-service grocery stores.

A CP from the Rockland County Health Department is assisting with food distribution as part of the food bank of the Hudson Valley’s Get Fresh Program. In April and early May 2020, more than 63,000 pounds of food were delivered to Rockland County. Fresh produce and dairy products were distributed to 20 food pantries, group homes, and food programs.

### Building on partnerships to create safe spaces for physical activity

CPs have worked in more than 200 communities to expand bike infrastructure, make streets and sidewalks safer for walking, and increase access to parks. For example, Common Ground Health invested heavily in expanding access to public spaces by advancing Play Walk, Safe Routes to Parks, and 10-Minute Walk to Park initiatives. Now more than ever, these outdoor spaces have helped residents to safely engage in physical activity at a distance during the pandemic. However, high-volume areas, or places restricted by right-of-way widths and other limited designs, continue to challenge pedestrians who are attempting to follow safe distancing guidelines. GObike Buffalo has helped to promote safe recreation and socially responsible active transportation by ensuring that bike shops are considered an essential business, launching a bike match program to connect those in need of a bike with others who have one to give, developing a tip sheet for municipalities outlining quick and inexpensive options for open streets and temporary “pop-ups,” and creating an outdoor opportunity index and map to identify areas where people may not have access to public spaces for recreation where they can maintain a safe social distance. GObike Buffalo is engaging residents as part of the Better Streets, Better Buffalo campaign to advocate for safe public spaces throughout the city.

### Working together to keep remote learners active

Before the pandemic, CPs of the Chautauqua Health Network made substantial strides in institutionalizing opportunities for physical activity throughout the school day. Since schools have closed and efforts have shifted to distance learning, administrators and teachers have worked to maintain academic standards. However, many schools partnering with the Chautauqua Health Network integrated the Daily Mile program, where kids ran a mile every day and recorded their mileage on an online platform; this has helped both teachers and students continue to track their progress during the pandemic. Now instead of logging progress at school, students are encouraged to log at home either online or on a worksheet.

### Collectively planning for the future

COVID-19 highlighted areas in need of intervention related to food access, physical activity, and active transportation. It has also served as a “forced pause,” offering CPs the opportunity to think strategically and creatively about how to build and strengthen partnerships, enhance communication channels, connect particular populations with resources, and advance efforts when the pandemic subsides. During several brainstorming calls, CPs shared strategies they were employing to move CHSC deliverables forward.

Many CPs are advancing their plans as intended through virtual meetings. For example, before COVID-19, the Chautauqua Health Network was in the process of developing a trail through Jamestown. In responding to physical distancing guidelines, the Network has continued moving forward through virtual meetings. The Buffalo Region CP is working with the statewide professional organization for physical and health education; local partners, including physical education and health teachers; and the local PBS (Public Broadcasting Service) station (WNED) to develop physical education and health lessons that can be aired on television. The CP reported, “We want to get physical education lessons out to students who don’t have internet access, but we also want to use this opportunity to advocate [for physical activity].” Several other CPs have used this time to develop and disseminate materials using data they have previously collected. The Genesee Valley Educational Partnership CP created a wellness policy implementation tool.

## Implications for Public Health

As indicated by the African proverb “It takes a village to raise a child,” it also takes the support of many to successfully implement PSEs. Similar to the CDC obesity prevention initiative (11) and project ACHIEVE (18), NYSDOH and CPs have identified OPCE as a valuable resource in providing or linking them to content experts, other practitioners, and resources related to their PSE initiatives. Findings from an evaluation of ACHIEVE (18) showed that the practitioners engaged in PSE-related work benefitted from the wide-ranging technical assistance that was provided by the National Association of Chronic Disease Directors and others. We are finding this also to be true in New York State, and the HCD approach to capacity building seems to be especially helpful during COVID-19.

The uncertainty, isolation, and anxiety of COVID-19 are real. As we navigate the pandemic, the goal of OPCE continues to be building capacity and strength at the individual and organizational levels so that CPs and their partners can continue to implement PSEs across their communities. The acts of regular discovery, definition, design, and implementation enable OPCE to provide responsive capacity building to CPs and their partners, especially during a pandemic such as we are now experiencing.

Finally, if there is a silver lining to COVID-19, perhaps it will include the priority we as a nation place on health and the significance of the environment in supporting healthy behaviors. Moreover, building capacity in low-income communities and communities of color should be prioritized, given existing racial and ethnic health-related disparities (25). In New York State, CPs will continue to engage with their school and community partners to ensure that PSEs are in place to increase access to nutritious foods and opportunities to be physically active. Using the 4 iterative phases of HCD may help organizations to be responsive to the constantly changing needs practitioners have when implementing PSEs. This iterative process may be especially helpful during crises like COVID-19. Given that CHSC is a project specific to New York State and that OPCE efforts were tailored to obesity prevention, more research and evaluation should be conducted to better understand how the use of HCD could support practitioners addressing other complex public health issues in the nation.
